# Affective Ratings of Pictures Related to Interpersonal Situations

**DOI:** 10.3389/fpsyg.2021.627849

**Published:** 2021-02-05

**Authors:** Wivine Blekić, Kendra Kandana Arachchige, Erika Wauthia, Isabelle Simoes Loureiro, Laurent Lefebvre, Mandy Rossignol

**Affiliations:** Department of Cognitive Psychology and Neuropsychology, University of Mons, Mons, Belgium

**Keywords:** database, valence-arousal, interpersonal violence, psychopathology, empathy

## Abstract

Many studies require standardized and replicable protocols composed of emotional stimuli. To this aim, several databases of emotional pictures are available. However, there are only few images directly depicting interpersonal violence, which is a specific emotion evocative stimulus for research on aggressive behavior or post-traumatic stress disorder. The objective of the current study is to provide a new set of standardized stimuli containing images depicting interpersonal situations (both positive and negative). This will allow a sensitive assessment of a wide range of cognitions linked to social interaction (empathy, perspective taking, traumatic experiences, etc.). To this aim, 240 participants rated the valence and arousal of 79 pictures collected from online sources in 2018. Results showed (1) a distinctive pattern of valence and arousal regarding the picture content and (2) specific associations between those two dimensions. Taken together, these results suggest a good reliability of the selected images. In conclusion, our study provides an open access set of recent pictures depicting interpersonal situations along with normative valence and arousal ratings, that are available for download from: https://osf.io/ak4m7/?view_only=None.

## Introduction

The study of human emotions is a core topic in behavioral and cognitive areas (Mauss and Robinson, 2009). For instance, several psychiatric conditions are characterized by a biased processing of emotional information (Bishop, 2007; Bardeen et al., 2013; Bullock and Bonanno, 2013; Patiny et al., 2015; Bradley et al., 2016). They are believed to cause and maintain the symptoms in time and to determine the pathology’s severity (Dalgleish et al., 2003). Authors are therefore trying to understand, prevent, and modify such biased processes by using standardized experimental procedures that involve emotional information. Such studies tried to approach real-life pathological behaviors (such as ruminations, craving, or hypervigilance) through laboratory responses toward specific stimuli. For example, while anhedonia in depression is assessed though reactivity toward positive stimuli, craving in alcoholism is explored through responses toward images of empty bottles, or social situations linked with drinking behaviors. In this context, the choice of proper stimuli in a laboratory cognitive task is a capital debate (Sheppes et al., 2015). Researchers have used different types of emotionally evocative stimuli, such as words, sounds, or pictures of faces or ecological scenes. First, studies testing these different types of stimuli suggest the existence of differences in the neural processing of written words and pictorial stimuli of emotional content. For example, pictures are better remembered than words (Quinlan et al., 2010), and generate higher arousal levels than written language (Bayer and Schacht, 2014). This might indicate that pictures are more biologically relevant than words and require less semantic processing (Soares et al., 2015). Second, since pictures require minimal linguistic knowledge (contrary to written or verbal stimuli), they are particularly suitable for cognitive research on affective processing (Hinojosa et al., 2009).

Laboratory cognitive task using emotional stimuli rely on a dimensional approach of the affective experience (Posner et al., 2005). This approach depicts that affective responses can be conceptualized and measured by both specific neural activations and self-reported subjective ratings on valence (i.e., the positive or negative affective experience) and arousal (i.e., the level of excitement induced by the stimuli) (Russell, 1980). In other words, each affective response is characterized by a specific activation on both valence and arousal systems. For example, fear is conceptualized as an emotional state that is the product of strong activation of negative valence, as well as strong activation of arousal systems (Bradley et al., 2001). Moreover, specific discrete emotions can be elicited by specific content categories. Indeed, researchers have observed that the semantic content of an emotional picture evoked specific functional changes in the brain. For example, a negative picture (with a low valence and high arousal) depicting a physical assault could result in an affective response of fear or anger, while a wound photography would rather elicit disgust (Mikels et al., 2005).

Several standardized databases of emotional evocative pictures were developed to provide standardized emotional evocative pictures, presenting ratings of valence and arousal, as well as semantic categories for discrete emotions. These databases, such as the International Affective Picture System (IAPS, Lang et al., 2008) or the Open Affective Standardized Image Set (OASIS, Kurdi et al., 2017), consist of colored photographs representing animals, objects or scenes that are either neutral or emotionally evocative. Each stimulus is accompanied by its standardized assessment in terms of valence (ranging from “pleasant” to “unpleasant”), arousal (ranging from “calm” to “excited”), and dominance (ranging from “influenced by the emotion evoked by the picture” to “in control of it”). Those dimensions have been assessed by the Self-Assessment Manikin (SAM, Bradley and Lang, 1994) or analog measure correlated to the SAM, specifically targeting the affective responses associated with the stimuli (“*How do you feel while viewing the picture*”) as opposed to the semantic knowledge about them (“*are the object or situations depicted good or bad?*”) (Hamzani et al., 2020). The normative rating procedure of emotional images started with the IAPS that has been conducted by Lang et al. (2008), among American College students. A total of 1,195 pictures were rated by approximately 100 participants. Subsequent studies have attempted to replicate the normative data of the IAPS for different age ranges, cultures and languages (Verschuere et al., 2001; Grühn and Scheibe, 2008; Drače et al., 2013; Soares et al., 2015; Bungener et al., 2016; Gong and Wang, 2016). It is important to note that these studies only evaluated the dimensions of valence and arousal, the third scale of “dominance” often being abandoned considering that this dimension presents a great inter-individual heterogeneity and only explains a small proportion of variance (Grühn and Scheibe, 2008). For this reason, this dimension was also not considered in this study. However, evidences of the cross-cultural, age and gender validity of the IAPS suggest that a replication of the previous rating protocol would be valid across different populations (Silva, 2011; Drače et al., 2013; Soares et al., 2015).

Even though those databases have allowed the publication of thousands of replicable studies among different populations and for different purposes, the domain of pathologies related to interpersonal violence has been put on the side. Indeed, among the semantic categories proposed by the IAPS and the OASIS, researchers can find pictures related to substances, medical conditions, animals, emotional states, objects, and so forth (Lang and Bradley, 2007; Kurdi et al., 2017). However, only few images are available in areas such as domestic violence, physical aggressions or robbery. Yet, they are specific triggers in conditions as post-traumatic stress disorders, psychopathy, delinquency, antisocial personality disorders and more generally in disorders sensitive to interpersonal violence (Kennedy, 2020). For example, psychopathy has been characterized by deficits in the recognition of facial emotions (Del Gaizo and Falkenbach, 2008) and distress among others (Kimonis et al., 2012) as well as difficulties to take the other’s perspective (Seidel et al., 2013) potentially leading to deficits in empathy (Drayton et al., 2018). While some of these studies have been conducted using standardized facial emotional stimuli (Mowle et al., 2019) such as in the NimStim database (Tottenham et al., 2009), these stimuli do not match the visual complexity of daily life situation and therefore, do not fully represent an ecological emotional processing (Sadeh and Verona, 2012). Therefore, researchers in the area of empathy related processes among this population had to either create unique interpersonal scenes through Adobe Photoshop (Seidel et al., 2013) or select them from the Internet (Jackson et al., 2006; Kimonis et al., 2006, 2012; Decety et al., 2013). As a consequence, research on the topic of interpersonal violence is often build with un-normative stimuli, complicating the replication and comparison of results on a world-wide scale (Sauer et al., 2014; Kennedy, 2020). This in an important issue considering that those violent-related behaviors or pathologies can hardly be candidates for ecological evaluation (which relies on naturalistic exposures to cues in their environment), they are almost only studied within laboratories.

Therefore, this study aims to create an open-access stimulus-set containing standardized images depicting interpersonal situations, both positive and negative. We selected 79 images depicting this theme and recruited 240 students for a norming study in which they have to provide self-reported subjective ratings of *valence* and *arousal*, as defined and measured in the IAPS. This study needs to be considered as a prerequisite for research in need of pictures related to interpersonal situations and should be replicated in clinical population. By using such an endorsed rating protocol, we aimed to provide researchers with a validated set of emotional pictures to complement those available in the IAPS (Lang and Bradley, 2007) and the OASIS (Kurdi et al., 2017).

## Materials and Methods

### Material

Two master-level psychologists selected the images using the Google Search tool. Pictures depicting interpersonal violence were selected by combining the following headings: physical aggression, assault, physical assault, terrorist attack, harassment, robbery. Pictures depicting positive interpersonal situation were selected using the headings: laugh, travel, friends, hiking, road trip, happiness. Pictures that (1) did not specifically focus on the semantic topics targeted; (2) were not available in high resolution (1,024 × 768); (3) were not on free access; (4) contained written information were excluded from the sample. If a disagreement existed, two Ph.D. students (first and second author of this paper) included or excluded the pictures based on the interpersonal character of the image. Finally, 44 pictures depicting interpersonal violence and 35 positive pictures were selected.

### Questionnaires

#### Self-Assessment Manikin (SAM, Bradley and Lang, 1994)

The SAM is a 9-point scale picture-oriented questionnaire; 9 representing a high rating on each dimension (i.e., high pleasure, high arousal), and 1 represents a low rating on each dimension (i.e., low pleasure, low arousal).

#### Anamnestic Questionnaire

Participants were asked to provide information regarding their age, gender, grade level, significant medical, or psychiatric condition, visual acuity (normal, corrected, impaired), and maternal language.

#### Population

In order to detect an effect size of Cohen’s *d* = 0.25 with 95% power (alpha = 0.05, one-sample case), G^∗^Power suggests we would need 175 participants. In prevision of dropouts, 240 French-speaking students between 18 and 25 years of age participated to the study in exchange for course credits. This specific age range was selected to replicate the original normalization study. Participants who reported significant medical or psychiatric condition, and who reported another maternal language than French were excluded from the study, as well as individuals who completed less than half of the task, resulting in a total sample of 196 participants (144 women - 52 men; mean age = 20.73; *SD* = 1.69).

#### Procedure

**FIGURE 1:**
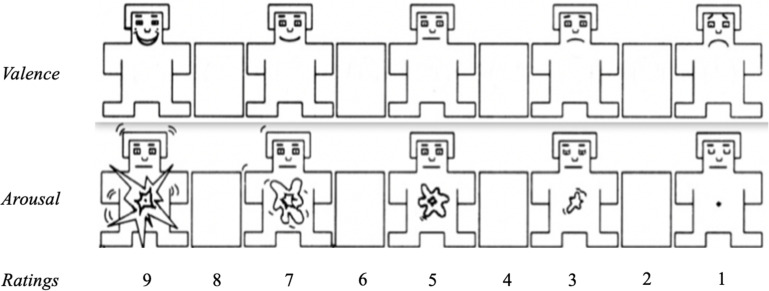
Subscales of the self-assessment Manikin used to assess valence and arousal in the present. The rating line was only presented during the instructions.

Study procedures were approved by the Ethics Committee of the University of Mons. Participants were gathered in a university amphitheater by groups of 30–50 and were separated from their neighbors by two empty seats. Two experimenters were present to give information on the study and to warn about the emotional content of the pictures. Once the information letter was read and the consent form signed, the instructors stated the detailed explanation of the normative procedure. The full instructions were given using a French translation of the directives given for the validation of the IAPS pictures (see Lang et al., 2008 for the detailed instructions). The instructors first explained to all participants the meaning of valence and arousal, and how to rate the pictures on their notebook. Instructions were given to specifically target the affective responses associated with each stimulus. Ninety-six pictures were presented on PowerPoint and displayed through a projector using the following sequence: a warning slide was displayed for 5 s, informing of the number of the picture to be rated. The picture itself followed and was shown for 6 s. Participants then had 15 s to rate the valence and arousal of the picture on a paper-pencil version of the SAM. The rating scales can be found in Figure 1, ranging from 1 to 9. Participants were asked not to verbally react to the images to avoid influencing their peers. Before starting the normative procedure, participants went through three practice trials, also proposed in the original study, in order to familiarize themselves with the rating procedure: one neutral with low arousal item, one positive with high arousal and one negative with high arousal (respectively, the IAPS slides #3181, #4420, and #7010). All participants rated the 79 pictures on both valence and arousal, resulting in a rating task of 45 min.

### Analyses

Analyzes were performed on SPSS software version 21. The normality of valence and arousal’s rating distribution was assessed with a Kolmogorov-Smirnov test. Between-groups comparisons were performed on gender using independent *t*-tests. Interrater reliability of such ratings was (1) ensured by an intra-class correlation coefficient (ICC) and (2) evaluated though the face validity of these valence and arousal ratings. We then conducted independent samples *t*-test to evaluate differences in valence and arousal ratings for positive and negative pictures. Finally, we performed linear and quadratic regressions on the data with the arousal mean as predictors and the valence mean. Indeed, these two constructs are not independent of each other among the IAPS pictures (Ueno et al., 2019). Scatterplots for valence (X) and arousal (Y) ratings form a U-shaped function, negative pictures being more arousing when their valence decreases, and positive pictures being more arousing when their valence increases. Pearson’s correlation coefficients (*r*) were also calculated to clarify the correlations between the valence and arousal ratings in our sample.

## Results

### Gender Effect

As our sample presented a higher rate of female participants, we firstly wanted to make sure that rating did not differed between gender. Independent *t*-tests confirmed that no significant differences between genders was present neither on the valence (*t* = −1.66, *p* = 0.100) or arousal (*t* = −1.29, *p* = 0.200) ratings. Thus, all further analyses were conducted using the full set of data combined.

### Ratings Distribution

The number of valence ratings provided for each image ranged from 188 to 196, with a mean of 194.34 ratings (*SD* = 1.51) per image. The number of arousal ratings provided for each image also ranged from 188 to 196, with a mean of 194.27 ratings (*SD* = 1.57) per image.

**FIGURE 2:**
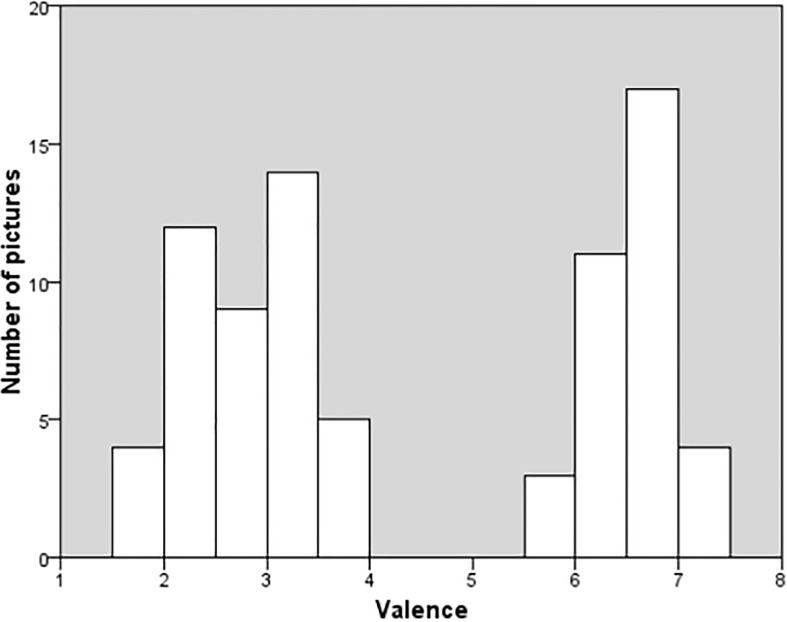
Distributions of image mean valence. The gap between 3.83 and 5.86 is related to the absence of neutral pictures.

The mean valence and arousal ratings and the corresponding standard deviations (*SD*) were calculated for each image. Valence ratings ranged from 1.91 to 7.17, showing good usage of a large range of the scale. The mean valence rating was 4.77, which is almost the theoretical mid-point of the scale. The distribution of the image mean valence is shown in Figure 1. Arousal ratings ranged from 3.32 to 7.27. Therefore, the range of arousal ratings was more restricted than the range of valence ratings. The mean arousal rating was 5.32, somewhat above the theoretical midpoint of the scale. This is not surprising considering that the presence of a majority of negative images in our study, who are known to be more arousing than positive ones. As we can see in Figure 2, we have a gap on valence rating between 3.83 and 5.86, as we did not used neutral images. Due to this gap, we separately analyzed positive and negative images instead of considering valence and arousal as a continuum.

For positive images (*N* = 35), valence ratings ranged from 5.86 to 7.17 and arousal ranged from 3.33 to 5.78. The median standard deviation was, respectively, of 1.36 and 2.27. A Kolmogorov–Smirnov test for uniformity confirmed a normal distribution of valence [*D*(144) = 0.818, *p* = 0.516] and arousal ratings [*D*(143) = 0.907, *p* = 0.384]. For negative images (*N* = 44), valence ratings ranged from 1.91 to 3.83 and arousal ranged from 4.59 to 7.27. The median standard deviation was, respectively, of 1.12 and 1.75. A Kolmogorov–Smirnov test for uniformity confirmed a normal distribution of valence (*D* = 0.77, *p* = 0.595) and arousal ratings (*D* = 0.68, *p* = 0.741).

### Reliability of the Ratings

For each dimension, inter-rater reliability was calculated though an intra-class correlation coefficient (ICC). To do so, we randomly generated a split half, calculated the correlation between the two halves, and took the mean of the correlation distribution as our reliability measure. For the valence dimension, the inter-rater reliability was excellent, R_val_ = 0.998 (*SD* = 0.001, range: *R*_min_ = 0.997 and *R*_max_ = 0.999). For the arousal dimension, the interrater reliability was somewhat lower but still outstanding, *R*_aro_ = 0.977 (*SD* = 0.008, range: *R*_min_ = 0.966 and *R*_max_ = 0.985).

Then, we evaluated the face validity of these valence and arousal ratings by probing which images received (1) the most highly positive and negative valence ratings, (2) the highest and lowest arousal ratings, and (3) the highest and lowest valence and arousal *SD*, indicating, respectively, low and high levels of agreement (Kurdi et al., 2017). The most positive valence rating (*M* = 7.17, *SD* = 1.34) was obtained for image I74, which depicts a breakfast moment during holidays, and the most negative valence rating was obtained for image I8 (*M* = 1.91, *SD* = 1.12), which depicts a physical aggression of a man against a woman. This last image also had the lowest valence standard deviation (1.12, *M* = 1.91), and image I38, which depicts a hunting scene between a man and a seal had the highest valence standard deviation (1.84, *M* = 1.9).

The highest arousal rating (*M* = 7.27, *SD* = 1.85) was obtained for image I8 previously described, and the lowest arousal rating (*M* = 3.33, *SD* = 2.02) was obtained for image I23, which depicts a young women taking a picture of a tree with her mobile phone. Image I72, which depicts two white males assaulting a third one next to a car had the lowest arousal standard deviation (1.75, *M* = 5.03), and image I45, which depicts Mickey and Minnie in Disneyland had the highest arousal standard deviation (2.72, *M* = 5.17). These values demonstrate face validity and confirm the soundness of our valence and arousal measures.

### Relationship Between Valence and Arousal

Independent samples *t*-test were used to evaluate differences in valence and arousal ratings. For the valence dimension, a significant difference in ratings between positive and negative pictures was reported, *t*(195) = 43.98, *p* < 0.001, 95% CI [3.59, 4.00], *Cohen’s D* = 3.135 (large effect size). Negative pictures were rated lower in the valence SAM scale (*M* = 2.8, *SD* = 0.82, min = 1.08, max = 5.31) than positive pictures (*M* = 6.76, *SD* = 0.64; min = 5.31; max = 8.73). For the arousal dimension, a significant difference in ratings between positive and negative pictures was reported, *t*(195) = 11.83, *p* < 0.001, 95% CI [-1.69, 4–1.09] *Cohen’s D* = 0.839 (large effect size). Negative pictures (*M* = 5.94, *SD* = 1.28; min = 1.95, max = 8.75) were rated higher in the SAM arousal dimension than positive pictures (*M* = 4.68, *SD* = 1.41; min = 1.08, max = 7.88). These results are highly similar to the corresponding results from the IAPS and the OASIS.

**FIGURE 3:**
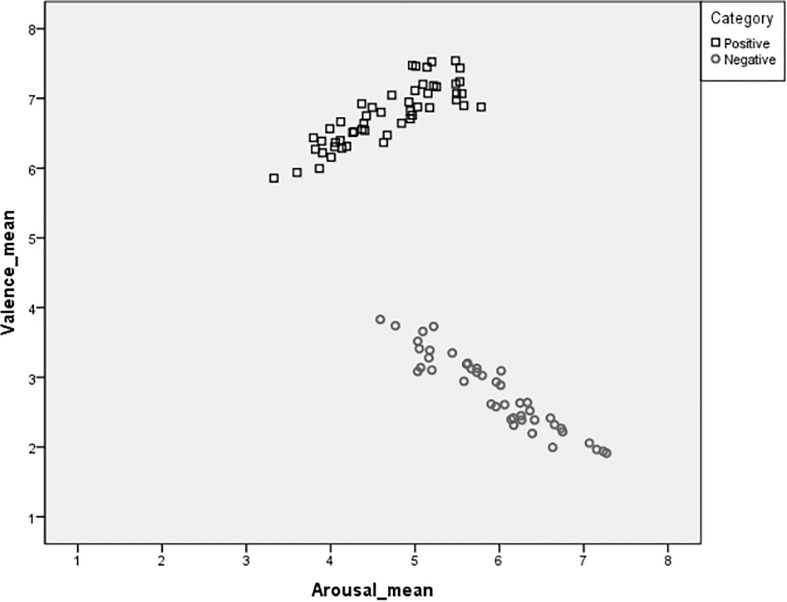
Scatterplots for valence and arousal ratings of the 79 pictures selected in the present study.

Valence and arousal ratings show a U-shaped bivariate distribution, such that arousal ratings are highest at the most positive and most negative levels of valence. Figure 3 presents a scatter diagram for valence and arousal ratings, divided in positive and negative pictures. As this study did not include neutral images, the top of the U-shape (corresponding to neutral ratings of valence along with low arousal ratings) is missing. However, the rest of the data behave as expected. To formally confirm this visual impression, linear and quadratic regressions to the data were performed, with the arousal means as predictors and the valence means and valence means squared as criterion variables. As expected, the quadratic regression provided the best fit to the data (*R*^2^ = 0.932) in comparison with the linear regression (*R*^2^ = 0.764).

Pearson’s correlation coefficients (*r*) were calculated to clarify the correlations between the valence and arousal ratings in our sample. For negative pictures, results showed that the valence ratings were negatively correlated with arousal ratings (*r* = −0.940, *p* < 0.001), such as pictures with low valence (negative) were more highly arousing. For positive pictures, results showed that valence ratings were positively correlated with arousal rating (*r* = 0.861, *p* < 0.001), such as pictures with high valence rating (positive) were more highly arousing.

## Discussion

This study aimed to provide a new set of emotional pictures accompanied by normative data to complement those available in the IAPS (Lang and Bradley, 2007) and the OASIS (Kurdi et al., 2017). Indeed, even though these databases cover a large range of semantic contents, specific research focusing on interpersonal violence has been left out in the past (Kimonis et al., 2012). Accordingly, negative pictures depicting interpersonal violence were evaluated and compared to positive pictures in terms of valence and arousal by a sample of young adults. The internal consistency results for the rating scales was proven to be high in this study, the Cronbach’s α coefficients for valence and arousal both being above 0.90. The subsequent analyses conducted on these dimensions showed that positive pictures were rated higher on the valence scale (*M* = 6.64), in comparison to the negative ones (*M* = 2.7). According to Grühn and Scheibe (2008), the valence cut-off using the SAM for a negative picture is inferior or equal to 4, whereas the valence cut-off for a positive picture is superior to 6. Given the means of valence for both our positive and negative categories, we can conclude that they have been correctly recognized and categorized as such. Consistently with previous studies, positive pictures were rated lower on the arousal scale than the negative ones.

Similar to the IAPS and OASIS, the mean ratings on the valence and arousal dimensions formed a normal distribution and presented a negative linear relationship with each other. This association between valence and arousal ratings was deepened and revealed a U-Shape, in such a way that highly positive and highly negative pictures were rated as more arousing. Therefore, we assume that the pictures validated in our study present the same characteristics as the one present in the IAPS. Importantly, the validation studies performed on the IAPS have firstly confirmed the cross-cultural validity of the IAPS, with only few differences found within the Asian population (Bungener et al., 2016). Specifically, erotic pictures tend to induce negative evaluation from Chinese females, while it was rated positively by the American original sample (Huang et al., 2015). Secondly, authors have highlighted differences in ratings induced by age. Studies have found that older adults (age from 65 and more) perceived positive picture as more positive and less arousing than younger adults, and negative pictures as more negative and more arousing than younger adults (Grühn and Scheibe, 2008). Despite those small differences, authors unanimously agree on the validity of the IAPS among different cultures and ages. In line, we assume that the pictures present in our study follow the same pattern.

Among the set of negative interpersonal pictures, a large range of arousal was reported, some pictures being rated quite low while others were rated high on the arousal scale. As a matter of fact, our database allows the users to select either low, mid or high aroused negative interpersonal situation that could be matched to specific conditions, from alexithymia to hypersensitivity. For instance, patients of several critical pathologies, such as post-traumatic stress disorder, are known to be too sensitive to handle highly arousal stimuli (Engel-Yeger et al., 2013). In line, it is to be noted that while gender did not have a significant effect among healthy controls in the present research, it might not be the case among specific pathologies. For example, it is expected that females with PTSD do not react the same way than males to negative interpersonal situation, or that males with an antisocial personality disorder will not provide the same ratings than females. To this aim, we provide in our supplementary material gender-specific ratings of valence and arousal for each picture. This will allow researchers to make an informed choice regarding the picture that can be selected for they own experiments.

This work should however be considered in light of several limitations. First, neutral images were not used. As existing database provided sufficient examples of such images, we focused on pictures depicting interpersonal situations. However, neutral pictures might have been an interesting comparison point. Researchers are invited to replicate the normative data present in this paper and add neutral images to complete existing analyzes. Second, some pictures present a higher standard deviation for valence and/or arousal, which reflects lower levels of agreements between raters. Academics that will use our pictures sample should be aware of such differences in standard deviation and carefully chose the pictures needed according to their own research questions and hypothesis. Third, age and educational background are limited in the present study. While previous research has proven a good consistency of affective rating across different age range, researchers working with elderly population might find different ratings. Indeed, as described above, a positive bias is often noted in this specific population. Finally, even though instructions were given in order to capture affective responses toward the situations depicted, it is possible that some participants were influenced in their responses by their own personal history or general stored representations about the valence of such pictures. In that case, the ratings could represent semantic valence and not affective responses as expected.

## Conclusion

The present study proposes an open-access online stimulus set specifically depicting interpersonal violence color images normed on two affective dimensions, valence and arousal. These stimuli include mid- to high-arousing images. Our results demonstrate the reliability of the selected images and provide a set of stimuli usable in a wide range of clinical and healthy population. We are hoping that this new dataset will be useful for researchers specifically interested in interpersonal violence triggers to avoid the previous flaws of un-normative protocols, therefore allowing a replication and comparison of their results on a world-wide scale. The stimulus set of 79 color images along with normative data is available at https://osf.io/ak4m7/?view_only=b1f31811d35e4b26a64eb6b65d01d467. The images can be downloaded, used, and modified free of charge for research purposes.

## Data Availability Statement

The pictures and normative data presented in this study can be found in online repositories. The names of the repository and accession link can be found below: https://osf.io/ak4m7/?view_only=None.

## Ethics Statement

The studies involving human participants were reviewed and approved by Ethics committee of the University of Mons. The participants provided their written informed consent to participate in this study.

## Author Contributions

WB designed the experiment. WB and KK stated on the inclusion or exclusion of the pictures if a disagreement existed. EW and IS were included in the statistical analyses of the manuscript. LL and MR supervised the project, gave feedback on the manuscript and suggested the investigation of several theoretical points (empathy processes, applicability, etc.). All authors discussed the results and contributed to the final manuscript.

## Conflict of Interest

The authors declare that the research was conducted in the absence of any commercial or financial relationships that could be construed as a potential conflict of interest.
